# Accounting for the dissipation of abiotic resources in LCA: Status, key challenges and potential way forward

**DOI:** 10.1016/j.resconrec.2020.104748

**Published:** 2020-06

**Authors:** Antoine Beylot, Fulvio Ardente, Serenella Sala, Luca Zampori

**Affiliations:** European Commission, Joint Research Centre, Via Enrico Fermi 2749, I-21027, Ispra, VA, Italy

**Keywords:** Resources, Dissipation, Loss, LCA, LCI, Impact assessment

## Abstract

The concept of resources or materials dissipation after their use in the technosphere has been increasingly considered in life-cycle based studies, applying Substance and Material Flow Analysis (SFA and MFA), Input-Output Analysis, and Life Cycle Assessment (LCA). However, there is currently no common understanding of what a dissipative flow is. This article first reviews 45 publications to describe the status of resource dissipation in life-cycle based studies, discussing how resource dissipation is usually defined, which temporal perspective is considered, which compartments of dissipation are distinguished, and which approaches (including the implementation of parameters) are considered to assess resource dissipation in a system. Moreover, this article proposes a comprehensive definition of resource dissipation, building from the literature review and focusing on abiotic resources. It then discusses this definition with respect to its potential implementation in LCA considering today’s existing Life Cycle Inventory (LCI) datasets and best practices. Overall it shows that the LCA framework may be well suited to assess abiotic resource dissipation. In particular i) the compartments of dissipation usually considered in the literature are covered in LCA, and ii) LCI databases could be a source of information to be further used to quantify a set of flows defined as “dissipative”, as commonly considered in SFA/MFA studies. However, major challenges are still faced before any potential routine implementation in LCA. The article accordingly discusses the potential way forward in the short-term (development and test of possible approaches), mid-term (towards satisfactory robustness, and consensus) and long-term (large-scale changes of LCI databases).

## Highlights

• Dissipation is a concept increasingly considered in LCA/MFA/SFA/IO Analysis.
• We review the way resource dissipation is addressed in 45 life cycle-based studies.
• Major differences arise in the approaches to account for this concept.
• We provide a comprehensive definition of abiotic resource dissipation.
• We discuss its potential implementation in LCA, as of today and in the future.

## 1 Introduction

The assessment of potential environmental impacts associated to abiotic resource use in Life Cycle Assessment (LCA) is a highly debated topic. So far, one of the major approaches to account for the impacts due to mineral resource use in the Life Cycle Impact Assessment (LCIA) step relies on the concept of depletion. The extraction of a resource from the Earth’s crust implies the reduction of the corresponding geological stocks, and is considered to subsequently contribute to this resource depletion. In particular, the so-called ADP (Abiotic Depletion Potential; Guinée et al., 2002; van Oers et al., 2002) model is currently recommended by the European Commission (EC) within the framework of the Product and Organisation Environmental Footprint (PEF/OEF) to assess the impacts due to mineral and metal resource use (EC, 2017; Zampori and Pant, 2019).

However, abiotic resources may remain in the anthropogenic system, although transformed, and may be available for further uses. Accordingly, several authors (Yellishetty et al., 2011; Klinglmair et al., 2014; Frischknecht, 2014; Schneider et al., 2011, Schneider et al., 2015; van Oers and Guinée, 2016) have discussed the possibility to consider also the amount of resources in the technosphere (e.g. in the form of scraps or waste) as part of the stocks potentially available in addition to geological stocks, and to include them in the calculation of characterization factors for assessing resource depletion. In parallel, as opposed to this concept of stocks of resources potentially available within the technosphere, the concept of resources or materials dissipation after their use in the technosphere has been increasingly considered in the fields of Substance and Material Flow Analysis (respectively SFA and MFA). Some authors have additionally called for considering this concept as well in LCA (Vadenbo et al., 2014), building on the foundations laid out by Stewart and Weidema (2005) and Heijungs et al. (1997). More recently, in the context of the Organisation Environmental Footprint Sector Rules (OEFSR) pilot, the need to move towards a dissipation concept has been highlighted (EC, 2018a), with a possible way forward as described in Annex V of the OEFSR on copper production (EC, 2018b). The dissipation of resources was identified as a promising concept, whose feasibility for implementation in LCA has been further discussed (Zampori and Sala, 2017). Moreover, the United Nations Environment Life Cycle Initiative task force also called for the consideration of dissipation in LCA, with effect on both inventories and impact assessment methods (Sonderegger et al., 2020; Berger et al., 2020). However, more than 20 years after “resource dissipation” was first mentioned as potentially applicable to assess the impact on natural resources in LCA, there is currently no common understanding of this concept, and no synthesis on the studies that have used it so far (Lifset et al., 2012; Zimmermann, 2017).

In this context, this article aims at i) describing the status of resource dissipation in “life-cycle based studies” in the literature (that is, studies that trace the flows of materials from their extraction up to their end-of-life), and to provide a definition building from this review; ii) reporting the key challenges that are still faced to enforce this concept in LCA; and iii) discussing the potential way forward in the short-, mid- and long-terms for implementation in LCA. The two next Sections (2 and 3) respectively review the concepts of “resources” and “resource dissipation”, considering “resources” in their broad sense, i.e. in particular encompassing abiotic (fossil and mineral) resources, biotic resources, water, soil and land. Instead, Sections 4 and 5 focus on the resources mostly addressed in the literature relative to resource dissipation, namely abiotic resources, to provide a definition for resource dissipation and to discuss both the key challenges it implies for implementation in LCA and the way forward. Even if limited to part of the resources only, these two sections are also intended to be a basis for potential generalization to other types of resources.

## 2 On the concept of “resources”

The term “resources” is used several times in ISO 14040 (ISO, 2006), which sets the principles and framework for LCA. In particular, the ISO standard states that “LCA addresses the environmental aspects and potential environmental impacts (e.g. use of resources and the environmental consequences of releases) throughout a product's life cycle”, yet without providing a definition for “resources”. More generally, in many publications (in a broad sense; e.g. reports, communications, scientific articles), the concept of resource is taken for granted so that a clear definition is not provided. This is for example the case in the United Nations’ milestone report on “Our Common Future”, which includes the strategic imperative of “conserving and enhancing the resource base”, overall pointing out that securing resources is a must for sustainable development, but without defining “resources” (UN, 1983).

In their discussion on mineral resources in LCIA, Drielsma et al. (2016) underline the critical need for appropriate definitions when models are constructed, subsequently recalling the traditional definitions utilized by leading geological institutions. Similarly, we consider essential to first raise the attention on some aspects of what a natural resource is, before we can define appropriately how it may be dissipated. We acknowledge that a huge number of publications have referred to this concept in several environmental, economic, social and law studies. Yet an exhaustive review of definitions of “resource(s)”, as available in the existing literature, is out of the scope of this study.

Since the relevance of the concept of “resources” for the following analysis, we traced back on a non-exhaustive list of definitions to exemplify some key elements usually conveyed by different authors when referring to “resource(s)”. A summary of such definitions is provided in the supplementary information (SI document 1, as derived from Ardente et al., 2019). Although very heterogeneous (as presented in studies and publications with very different purposes), these definitions seem to converge to a common point: a “resource” is considered as such when it has an intrinsic “value” or “utility” (i.e. by providing a certain function) for a certain subject (generally humans, in the common anthropogenic perspective). This value or function is not exclusively “economic” but it can range from the satisfaction of specific needs (e.g. the properties of a material for a production process) to the contribution to the overall human well-being (e.g. through the “cultural value” of resources). These considerations subsequently imply that input/output flows occurring in a life cycle inventory do not necessarily relate to “resources”, in case these flows do not deliver any function or utility to the system while incidentally occurring in the process.

## 3 Resource dissipation in life-cycle based studies: status, from definition to implementation

This section firstly discusses the concept of resource dissipation as defined and implemented in the existing life-cycle based studies. These studies include MFA, SFA, LCA, and IO (Input-Output) Analysis. MFA, SFA and IO Analysis are closely interconnected: MFA is a general term including SFA, while IO Analysis can be used as a basis for Material Flow Analysis (so-called IO-MFA). In the following, the terms MFA and SFA are used when referring to the original developments in the field, while the term IO corresponds to any study based on IO Analysis, including what some authors refer to IO-MFA. This distinction enables to clearly identify IO Analysis as an approach which enables to account for both economic and physical exchanges in an economy and to allocate pressures to the environment (including resource extraction) to the final demand for goods and services.

The following discussion is based on a literature search performed through Scopus, online database of peer-reviewed scientific publications in English. Two keywords were combined in the query, and screened within title, abstract and keywords: firstly, one keyword related to the concept of dissipation (either “dissipation” or “dissipative”), and secondly one keyword referring to the method implemented (either using their entire name or only their acronyms; see SI document 2 for the complete list of keywords used for the search). As a complement, several articles and reports that have not been identified through this search process, but that we considered of particular relevance, have been additionally selected and reviewed. On the contrary, a first screening of this full set of references has enabled to exclude some articles, when the concept of dissipation was treated marginally. Overall, the following analysis is based on 45 publications reviewed (see SI document 2 for a complete list of publications reviewed and pieces of information drawn from the review process, on which the following discussion is based).

### 3.1 Scope of the studies

**Fig. 1:**
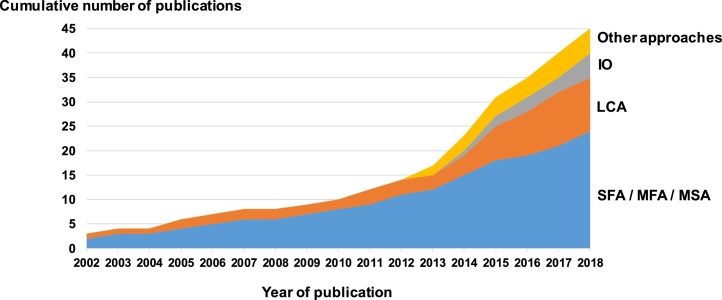
The 45 life-cycle-based publications dealing with the concept of dissipation considered in the review, by type of method implemented and year of publication.

Most studies in the review aim at the analysis of flows and stocks of materials, applying standard Material Flow Analysis, sometimes targeting substances (SFA) or systems (MSA) instead of materials in a general sense. Overall, MFA (including SFA and MSA) is the method implemented in more than half of the cases (24 out of 45). The share of MFA studies dealing with the concept of resource dissipation even represented approximately 80 % of the articles published from 2002 to 2012 (Fig. 1). From 2012 to 2018, a larger proportion of LCA, IO, and other approaches have tackled this issue as well, in particular highlighting the growing interest of the LCA-community on this topic. Moreover, 69 % of the publications apply an approach to a case study (in most cases namely applying MFA to a set of resources), while the remaining share aims at methodological developments. In particular, the majority of publications with LCA as the supporting method (9 out of 11) relate to methodological developments with respect to the accounting of resource use in the impact assessment.

Overall, the reviewed studies dealing with the concept of dissipation mainly target abiotic resources. Most studies explicitly target a defined set of chemical elements, essentially metals. This is for example the case for most MFA studies, which aim at quantifying the flows and stocks of some given elements, in particular metals (e.g. zinc; Spatari et al., 2003; Tabayashi et al., 2009; Guo et al., 2010) but also other elements (e.g. fluorine, Villalba et al., 2007). In addition, a number of studies also apply or discuss the concept of dissipation with respect to:

- Other abiotic resources, such as fossil fuels (Krausmann et al., 2018; BIO by Deloitte, 2015) and non-metallic minerals (e.g. Stewart and Weidema, 2005), including aggregates (BIO by Deloitte, 2015);
- Biotic resources (e.g. wild or domesticated plants and animals in Stewart and Weidema, 2005; food for humans or feed for livestock, and biomass for thermal conversion in Krausmann et al., 2018).

Finally, in the context of discussions on the AoP natural resources for application to LCIA, several authors also discuss, or refer to, the concept of dissipation with considering a broader scope of resources in their study, including e.g. water, land, soil and water surface (Stewart and Weidema, 2005; Sonderegger et al., 2017; Sonnemann et al., 2015).

### 3.2 How “dissipation” is referred to? Commenting the lexical field used

In the reviewed publications, the concept of dissipation is analyzed and discussed with predominantly employing the term “dissipation” and its derivate terms: “dissipated” and “dissipative”. In many MFA case studies aiming at the assessment of metals stocks and flows, authors refer *i)* to the “dissipation of” the metal under study (e.g. dissipation of chromium in Johnson et al., 2006; indium dissipation in Stamp et al., 2014), *ii)* to the metal “dissipated” (e.g. copper dissipated in Spatari et al., 2005; germanium dissipated in Licht et al., 2015; alloying elements dissipated in Ohno et al., 2015), and *iii)* to the “dissipative” metal (dissipative zinc in Tabayashi, 2009; dissipative lead in Liang and Mao, 2014). When authors refer to dissipation in a more general sense, in particular tackling more than one resource either in a case study or in a methodological discussion, they often refer to “dissipative flows” (Lifset et al., 2012; Thiébaud, 2018; Müller et al., 2014), also termed “dissipation flows” (Johnson et al., 2006), or “dissipative losses” (e.g. Rydh and Karlstrom, 2002; Spatari et al., 2002; Villalba et al., 2007; Guo et al., 2010; Ziemann et al., 2012; Zimmermann and Gößling-Reisemann, 2013; Kral et al., 2013; Licht et al., 2015; Zimmermann, 2017), sometimes using both interchangeably (Spatari et al., 2003; Ciacci et al., 2015; Kovanda, 2017). It is noteworthy that the term “loss” is also referred to in many publications without being directly associated to “dissipation” or “dissipative”. A clear connection is however often made between the two terms: for example, Stewart and Weidema (2005) define “resource dissipation” as a “loss of resource”, while Schneider et al. (2015) refer to “loss” of resources as a consequence of “dissipation”. Overall, two main cases can be distinguished:

a) for some authors, the concept of loss is intended to be broader than the concept of dissipation, that is “losses” account for, without being limited to, “dissipative flows” (e.g. BIO by Deloitte, 2015; Duygan and Meylan, 2015; Nakamura et al., 2014). For example, BIO by Deloitte (2015) define “losses” as the sum of three terms: *i)* outputs from the value chain (elements exiting the value chain “as impurities, non-functional by-product, dissipation…”), *ii)* in-use dissipation and *iii)* non-functional recycling;
b) on the contrary, other authors consider “dissipation” as a concept broader than “loss”. In this respect, Takeyama et al. (2016) evaluate the dissipation of chromium and nickel by summing the shares “lost” due to “physical and quality losses”. Moreover, Laner et al. (2017) refer to losses regarding different types of flows (e.g. “losses of phosphorous to landfills”) while the term “dissipation” refers more generally to a reduction in concentrations in the system, including due to “losses”.

Moreover, it is worth noting that whereas most authors refer to quantities of materials when using the term (dissipative) “loss”, others instead/also refer to losses in terms of quality (Paraskevas et al., 2015; Takeyama et al., 2016) or functionality (Stewart and Weidema, 2005; Sonderegger et al., 2017). Finally, several publications (e.g. Ziemann et al., 2012; Ciacci et al., 2015) include, or sometimes even focus on, “dissipative use”, also termed “in-use dissipation” or “dissipative applications”, which are described as specific uses or applications of the resource that directly lead to dissipation.

### 3.3 What is “resource dissipation”? One term, several definitions

Among the 45 publications considered in this review, 15 (33 % of the total) provide some definitions, most often explicit, of the concept of dissipation or of related terms. Table 1 lists the different definitions (in one case a definition was used in two publications). Instead, the remaining 30 publications do not explicitly define what they intend to capture as “dissipation”. The definition of dissipative flows is in these cases essentially implicit, with authors primarily classifying some flows as dissipative ‘*per se’* (i.e. considering the concept as self-explaining and not necessitating further clarification).

**Table 1:** “Resource dissipation” in the literature: a list of definitions.

Authors	Definitions
Stewart and Weidema (2005)	Resource dissipation is defined as “the loss of resources from the technosphere in such a way that it is not possible to recycle them back into the technosphere.”
Schneider et al. (2011)	"Dissipated stock is the amount of a resource that has been returned to nature in a form that makes recovery almost impossible"
Lifset et al. (2012)	The definition distinguishes between "dissipative releases, or releases from products that are not easily recovered or recycled; and dissipative uses, or those uses of a substance where the dissipative release is intentional"
Ziemann et al. (2012)	The definition focuses on dissipative use/applications. "A use or application of a metal is called dissipative when the metal is dispersed or scattered during the phase of use, making it exceptionally difficult and costly to recycle".
Zimmermann and Gößling-Reisemann (2013) and Zimmermann (2017)	“Dissipative losses for metals” are defined as “losses of material into the environment, other material flows, or permanent waste storage that result in concentrations in the receiving medium such that a recovery of these materials is technically or economically unfeasible."
Johansson et al. (2013)	"Dissipated metal resources […] represent the share of employed metals that has been dispersed to the surrounding environment (land, sea, air or even space), often due to friction or turbulence such as copper corrosion from the roofs, metal leaching from landfills or zinc emissions from brake linings […]. Another type of dissipation is debris. While space debris primarily consists of abandoned space bodies […], marine debris tends to originate from human mishandling of discarded items such as waste escaping from landfills […]."
Kral et al. (2013)	"Dissipative material losses cover point and diffuse emissions with no option for recovery."
Müller et al. (2014)	The authors of this review article first refer to Ayres (1994): the category "metals that have been irrecoverably dissipated into soil, groundwater, or surface water" accounts for "material dissipation"; “materials are recycled or reused if economically and technologically feasible, otherwise they are eventually dissipated". Moreover, the authors mention that in the full set of studies they have reviewed, “dissipative flows” are generally described as in Ayres (1994) or referred to “as emissions, loss flows, stock leakage or specific flows to landfills or the environment”.
Stamp et al. (2014)	"Dissipation is defined as a dilution to the extent that recovery is impossible with known technologies."
BIO by Deloitte (2015)	The authors state that losses are composed of: i) Output from the value chain: "annual quantity of the element exiting the value chain (as impurities, non functional by-product, dissipation, …)"; ii) In use dissipation: “refers for example to: a loss of zinc due to corrosion of zinc coating on steel, a loss of copper due to spread of copper sulphate as a fungicide"; iii) Non functional recycling: "refers to recycling in which the element in a discarded product is collected and incorporated in an associated large magnitude material stream. This represents the loss of its function as it is generally impossible to recover it from the large magnitude stream"
Ciacci et al. (2015)	"dissipative losses are the flows of materials from the anthroposphere (i.e., human systems) to the biosphere (i.e., environment) in a manner that makes their future recovery extremely difficult, if not impossible"
Sonnemann et al. (2015)	A resource is “dissipated” when it “is made unusable as such for future users”
Kovanda et al. (2017)	The dissipative flow account consists of: - "Dissipative use of products" which comprises “materials released into the environment on purpose in order to increase the production capacity of agricultural land, for instance. […] Dissipative use of products for the Czech Republic comprised consumption of mineral fertilizers […], manure […], pesticides […], seeds used for sowing […], composts, use of sewage sludge as fertilizer […] and consumption of thawing materials" - and "Dissipative losses”, which “include corrosion and abrasion of products and infrastructures, leakages, emissions from the use of solvents, etc."
Sonderegger et al. (2017)	Commenting articles in their discussion of the literature, the authors state that "If the natural resource is dissipated into concentrations that are below a threshold that allows for recovery, it is lost and the stock decreases." Later in their discussion, they mention that: "Natural resources and (raw) materials are lost if the required qualities for their functionality are lost (e.g., through dissipation).”

Among the 14 definitions, 9 use the term “recover” (or its derivate terms, such as “recovery”), and 5 the term “recycle” (instead of, or as a complement to, the term “recovery”). Dissipation of a resource is therefore primarily related to the difficulty, or even to the impossibility, to recover it. On the one hand part of the authors consider that, even if the resource is recoverable, it may be set to be dissipated in case of an extreme / exceptional difficulty to recover it. Accordingly dissipation is said to occur when the recovery or recycling is not easy (Lifset et al., 2012), “exceptionally difficult and costly” (Ziemann et al., 2012), “extremely difficult, if not impossible” (Ciacci et al., 2015) or “almost impossible” (Schneider et al., 2011). On the other hand, other authors clearly mention the impossibility for the resource recovery: the recovery or recycling is said to be “not possible” (Stewart and Weidema, 2005), “impossible” (Stamp et al., 2014) or “unfeasible” (Zimmermann and Gößling-Reisemann, 2013; Zimmermann, 2017), while some other authors additionally mention the terms “irrecoverably” (Müller et al., 2014), “no option for recovery” (Kral et al., 2013) and “below a threshold that allows for recovery” (Sonderegger et al., 2017) in their definition of dissipation. Finally, in some cases (e.g. BIO by Deloitte, 2015; Kovanda, 2017) authors provide partial definitions in which different types of dissipation are listed and supported by an extensive list of examples.

### 3.4 Which temporal perspective to assess dissipation? On the static/dynamic nature of dissipation

Several of the publications considered in the review implement a dynamic modelling of flows and stocks, for example considering assumptions on the implementation of improvements in technology (Nakamura et al., 2014). Moreover, regarding the assessment of these flows as “dissipative” or on the contrary “non-dissipative”, several authors refer to temporal aspects directly within the definition they provide for the concept of “dissipation” (Table 1), or as complements to this definition. In particular, Ciacci et al. (2015) define dissipative flows as those for which “future recovery [is] extremely difficult, if not impossible”. Similarly, Stewart and Weidema (2005) refer to “resources made unavailable […] for any foreseeable future use by society”, while Sonnemann et al. (2015) consider a resource as dissipated when it “is made unusable as such for future users”. Moreover, Zimmermann and Gößling-Reisemann (2013) add a "dynamic element” in their definition of dissipative losses ("losses that must be considered dissipative today might be less dissipative in the future"), which they link to the time-dependent technical and economic feasibilities of recovering a material, in line with some other authors (e.g. Lifset et al., 2012). They additionally mention the residence time as a qualitative parameter to classify dissipation types, with “long residence times […] increasing the severity of dissipation" (Zimmermann and Gößling-Reisemann, 2013). Yet, it is noteworthy that the majority of authors (69 % of publications) on the contrary do not refer to any temporal aspect in their approach to dissipation. Moreover, even in the cases where these aspects are mentioned, this is primarily done in very general terms. In particular, none of the publications reviewed explicitly mentions a given (valued) temporal perspective, for application to the quantification of dissipative flows.

### 3.5 Where are resources dissipated? Distinguishing three compartments of dissipation

**Fig. 2:**
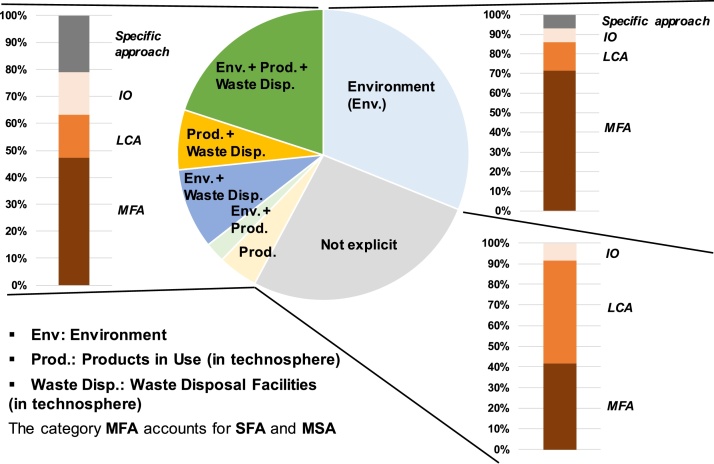
Where are resources dissipated? Shares of compartments of occurrence in the 45 publications reviewed, as a function of the method implemented.
- final waste disposal facilities (in technosphere), considered in 36 % of studies. This in particular corresponds to landfills and tailings management facilities (e.g. critical metals with a share dissipated to slags disposed of in landfills; Thiébaud et al., 2018);
- products in use (in technosphere), considered in 33 % of studies. This category corresponds to two main types of flows. Firstly, for some authors (e.g. BIO by Deloitte, 2015; Paraskevas et al., 2015; Takeyama et al., 2016; Laner et al., 2017), this category aims at accounting for “dissipation” or “losses” due to non-functional recycling, that is the “collection of old metal scrap flowing into a large magnitude material stream, as a “tramp” or impurity elements”, representing the “loss of its function” according to the United Nations Environment Programme definition (UNEP, 2011). For example Takeyama et al. (2016) state that nickel and chromium used in special (alloy) steel are expected to be dissipated when recycled to ordinary steel. Yet, it is noteworthy that some other authors instead exclude non-functional recycling as a form of dissipation (e.g. Ciacci et al., 2015). Secondly, the category “products in use” aims at accounting for dissipation (dispersion/dissolution) in products (e.g. indium used in solders and alloys; Licht et al., 2015), as a driver of subsequent dissipation later in the life cycle (e.g. “at the end of life” as in Licht et al., 2015; and/or through emissions to the environment during the product use; see also Zimmermann and Gößling-Reisemann, 2013 and Zimmermann and Gößling-Reisemann, 2014; Duygan and Meylan, 2015; Ohno et al., 2015). Such an accounting of dissipation in products in use however overlaps with the dissipation in final waste disposal facilities and emissions to the environment as considered by some other authors (and as listed above). Finally, as a specific additional type of dissipation in products in use, Zimmermann and Gößling-Reisemann (2013) and Zimmermann (2017) consider the impossibility to access the material embodied in a product in use, introducing the concept of short and long residence time in their discussion of resource dissipation.

Among the publications considered in this exercise, 73 % account more or less explicitly for dissipative flows to (or within) one of the three following compartments:

- environment, which relates to what is usually called “emissions to the environment” in MFA and LCA studies. For example, emissions of copper associated with its mining and production and with its use in specific applications (e.g. pesticides, fertilizers, fireworks, brake pads, etc.) are considered to be dissipative flows to the environment (Lifset et al., 2012). Environment is the compartment mostly addressed in life-cycle-based studies dealing with the concept of dissipation (considered in 62 % of studies, including 31 % as a single compartment; Fig. 2);

Among the publications that consider emissions to the environment as the only dissipative flows, two thirds relate to MFA (including SFA and MSA) studies (Fig. 2). Linking this result with the trend of Fig. 1, one observes that the life cycle community has generally considered in the past that dissipative flows were restricted to emissions to the environment. In their original developments, in the early 2000s, SFA studies also sometimes reported the flows of materials or substances to landfills and tailings, nevertheless without necessarily associating them with the concept of “dissipation” (e.g. Rechberger and Graedel, 2002; Rydh and Karlström, 2002; Spatari et al., 2003, 2005).

On the contrary, the share of each type of method is relatively more balanced regarding the studies accounting for dissipation in products in use and in waste disposal facilities (Fig. 2). The recent increasing interest of IO and LCA communities on the concept of "dissipation”, as observed in Fig. 1, has tended to reduce the share of “environment” as the only compartment considered for dissipative flows. Also, a number of recent SFA studies have considered compartments beyond environment when referring to dissipation (e.g. Licht et al., 2015; Ciacci et al., 2016; Thiébaud et al., 2018) contrarily to most of the SFA studies performed in the early 2000s. Finally, LCA is the method for which the type of compartments is the least explicitly addressed (Fig. 2): a number of LCA publications mention the concept of dissipation when discussing the impacts of the studied system due to resource use, yet without referring where these dissipative flows occur.

### 3.6 Which approaches are implemented to assess “resource dissipation” in a system?

Most authors define a set of flows that they consider “dissipative”, and then quantify them based on different types of data (statistics, process data, assumptions, etc.). On the one hand, some authors provide some or even several examples of dissipative flows, considered as self-explanatory and related to a common understanding of the dissipation concept, e.g. relating to the use of minerals in fertilizers (Zimmermann and Gößling-Reisemann, 2013; Ciacci et al., 2016; Ciacci et al., 2015; Kovanda, 2017; Laner et al., 2017; Krausmann et al., 2018), to the dissipation of certain metals when used in alloys (Zimmermann and Gößling-Reisemann, 2013; Licht et al., 2015; Ohno et al., 2015; Paraskevas et al., 2015; Takeyama et al., 2016; Nakamura and Kondo, 2018), or due to corrosion or abrasion (Schneider et al., 2011; Johansson et al., 2013; Kral et al., 2013; BIO by Deloitte, 2015; Ciacci et al., 2015; Kovanda, 2017). On the other hand, some authors use a generalized approach to classify dissipative flows, based on simplified assumptions. For example it is assumed that: all the flows to the environment (Arvidsson et al., 2011; Spatari et al., 2005; Johnson et al., 2006), or flows to tailings and slags (Liang and Mao, 2014) or flows to landfills (Zimmermann and Gößling-Reisemann, 2014) are dissipative. Finally, as a generalized approach at a more aggregated level, Vadenbo et al. (2014) suggest calculating the dissipative use of resources in LCA as “the difference between the amounts of resources extracted and recycled”.

However, in none of these studies the rationale for flagging flows as “dissipative” is quantitatively discussed, in particular entailing the risk of being non-exhaustive. Considering the definitions provided in the literature (Table 1), we may assume that this classification is based on the authors’ own evaluation of the difficulty, or even the impossibility, to recover the resource considering the flows at stake. Only a few authors mention parameters and thresholds to clearly distinguish dissipative flows from non-dissipative ones. Stewart and Weidema (2005) in particular mention concentration and mineralogy regarding metallic minerals, and particle size regarding non-metallic minerals, as parameters to assess dissipation in light of “ultimate quality limits”. Zimmermann and Gößling-Reisemann (2013) and Zimmermann (2017) mention concentration as a determining parameter in their definition of dissipative flows (Table 1), while Vadenbo et al. (2014) refer to recovery costs and resource concentrations as potential criteria to draw the boundary between borrowing and dissipative uses. Finally, Rechberger and Graedel (2002), Laner et al. (2017) and Thiébaud et al. (2018) are the only authors implementing a quantitative approach to case studies, namely using Relative Statistical Entropy (RSE) to express the ability of a process or of a whole system to dissipate a resource, as also mentioned in UNEP (2013). Yet this approach has not been used to distinguish dissipative flows from non-dissipative flows (that would be both expressed in mass terms), but rather to quantify dissipation along a system (using RSE as the metric).

## 4 Resource dissipation: a definition, and the key challenges it implies for implementation in LCA

This section firstly provides a definition for resource dissipation, building from the literature review and specifically focusing on abiotic resources (that is, fossil and mineral resources). Then, the concept of dissipation is discussed with respect to its potential implementation in LCA, distinguishing three determining features as considered in the above literature review: the temporal perspective, the different compartments towards which resources are considered to be dissipated, and the approach implemented to assess dissipative flows in the system under study.

### 4.1 Terminology and definition

The literature review highlighted the absence of a common definition of resource dissipation across all reviewed studies, with relatively large deviations from one study to the other. In this context, we propose a comprehensive definition for dissipation of abiotic resources, building on several definitions in the literature, to reflect relevant features as discussed in previous sections:

*Dissipative flows of abiotic resources are flows to sinks or stocks that are not accessible to future users due to different constraints. These constraints prevent humans to make use of the function(s) that the resources could have in the technosphere. The distinction between dissipative and non-dissipative flows of resources may depend on technological and economic factors, which can change over time.*

In particular, we have chosen to use the terms “dissipative flows” instead of generally referring to “dissipation” or “losses” (and their derivate terms, such as “dissipative losses”), primarily because they appear more connected to the topic addressed (resource dissipation) than any reference to “losses”. Moreover, the focus on flows is in line with the core practice of LCA of investigating exchanges of products and elementary flows between unit processes in the technosphere and the environment. Yet, we acknowledge that there may be some cases where the use of the term “loss” may be considered more understandable and therefore still appropriate, e.g. when communicating LCA results to a non-technical target audience.

When putting this definition in perspective with the above discussions on the concepts of “resources” and “dissipation” as in the literature, it is noteworthy that it refers to:

- the function a resource may have. For example the mass of any metal along the life cycle of a system remains constant (atoms of metals never “disappear”), and as such still hold a potential value for humans. Yet metals (considered here as “resources”) can be dissipated, whenever the functions these metals can have for humans in the technosphere are rendered inaccessible to future users along the life cycle of a system;
- the temporal dimension (mentioning “not accessible to future users”, “which may change over time”), therefore making the timeframe an element to be considered when quantifying resource dissipation;
- “flows to sinks or stocks”, therefore implicitly encompassing flows to the three compartments most commonly distinguished in the literature: environment, products in use and waste disposal facilities;
- “different constraints” which include, but are not limited to: change in physico-chemical properties; low concentrations and large spatial spreading; complex chemical and mineralogical compositions (e.g. presence of contaminants); heterogeneity and limited knowledge on sinks composition and localization which hinder the chance of their recovery;
- “technological and economic factors” as potential determinants to distinguish “dissipative flows” from “non-dissipative flows”. Yet this definition is also open to a purely physical understanding of the concept of dissipation, which could e.g. consist in directly identifying dissipation to entropy/exergy changes along the system under study, therefore beyond considering such entropy/exergy changes as markers of dissipation.

### 4.2 Which temporal perspective to assess resource dissipation in LCA?

The reference to “future users” and to time-dependent (technological and economic) factors in this definition implies that, when assessing resource dissipation over the life cycle of products and systems, the timeframe considered should be *i)* determined when defining the scope of the analysis (based on the answer to the question: which future users are considered in this assessment?) and *ii)* explicitly reported. This timeframe potentially has key implications on the technological and economic capacity to make use of the function of a resource, and subsequently on the assessment of dissipative and non-dissipative flows in a system.

Despite a precise specification of the timeframe to be considered is out of the scope of this article, it is noteworthy that:

- A very short timeframe would consider recycling performances at the time of the assessment and would assume that all the resources ending to final sinks (e.g. landfills, tailings), and not currently recovered, are dissipated. Such timeframe would be representative of the current situation (by definition), and would potentially convey a relatively high precision in the modelling of resource dissipation thanks to good data quality. However, it would disregard the fact that part of these sinks could be potentially exploited in the future (e.g. landfill mining), with a certain efficiency (therefore potentially lowering the accuracy of the modelling);
- A very long (or even infinite) timeframe could imply the assumption that, regarding any flow of resource currently unrecovered, there is a chance that future technological improvements will make it potentially functional again for humans. However, this chance is always mainly theoretical and very difficult to assess “a priori”, especially concerning the efficiency of processes and the characteristics of the recovered materials.

In the context of LCA, the timeframe considered to assess resource dissipation needs to be consistent with the goal and scope of the study, with potential influence on both the inventory and the impact assessment steps. In the new approaches to account for resource dissipation in LCA, several timeframes could be set for further choice and use by LCA practitioners, as is the case for some other impact categories.

### 4.3 Which compartments to assess resource dissipation in LCA?

The definition of resource dissipation broadly encompasses “flows to sinks or stocks that are not accessible to future users”. Accordingly, it includes emissions to the environment, flows to products then used in the technosphere and flows to waste disposal facilities, under two conditions: i) they are flows of resources and ii) they are made inaccessible to future users. For certain flows it could be not straightforward to define if this is an emission to the environment or within the technosphere. For example, copper used as pesticides and spread in agriculture could be considered as dissipated in the environment (as air emissions) or in the technosphere (as contaminant in a cultivated field). Actually, whatever the compartment of occurrence, the dissipative flows could be assessed considering a common temporal perspective (as discussed in the previous Section, 4.2) and a common set of parameters (as discussed in the next Section 4.4). Yet specifying the compartment can be relevant to better understand where dissipation occurs in a system (“what ends where”), and also to differentiate among “dissipative” or “non-dissipative” flows based on general, shared, rules (e.g. setting by default that all emissions to the environment could be considered dissipative).

The LCA framework and the current LCI databases overall distinguish emissions to the environment and products end-of-life (including recycling and waste disposal), in particular including some data that could be used to support the assessment of resource dissipation. Firstly, emissions to the environment are usually traced and documented in LCIs. These data, in mass units, could be considered as a basis to identify and quantify resources dissipated to the environment. However, not all emissions to the environment are necessarily dissipative flows of resources. Only the emissions of resources (which have held a function and/or an instrumental value along the system life cycle, by definition) can be potentially dissipative. For example, part of the heavy metals embodied in copper mineral ores which are displaced from the underground to other compartments through the process of copper concentration (e.g. to tailings or to air, as dust emissions from the process) are not exploited and do not act as “resources” along the system. They are potentially relevant regarding toxicity aspects (Beylot and Villeneuve, 2017), but could be disregarded with respect to their contribution to resource dissipation.

Moreover, LCA accounts for the end-of-life of the product or system under study, and of all the products and systems interlinked with it. This firstly implies accounting for the generation of waste and their recycling at the end-of-life. If possible, this analysis should also evaluate the degradation (“downcycling”) of the recycled materials’ functions (suggested, for example, as best practice by the ILCD Handbook; EC-JRC, 2010). The estimation of the “degradation” of resources (i.e. the changes of their functionality due to e.g. non-functional recycling, in quantitative and qualitative terms), have been used so far in LCA and Ecodesign studies to quantify potential environmental credits to recycling (Allacker et al., 2014; Ardente and Mathieux, 2014). These could be additionally used as a basis to estimate the mass of resources dissipated in the products in use within the technosphere. Further developments would however be necessary to systematically identify the cases for which the degradation of a resource is such that it is “dissipated” (e.g. setting appropriate metrics and thresholds). Moreover, LCA accounts for flows to waste disposal facilities (including in particular municipal solid waste, bottom ashes or slags landfilling, and waste from mineral processing disposal in tailings disposal facilities). Currently the LCI modelling primarily aims at providing the corresponding emissions to the environment (e.g. under the form of leachates). As best practice these emissions are derived considering both the mass and composition of the waste disposed of (see e.g. Doka, 2003). Yet, the information on the elemental composition of waste, used to calculate these emissions to the environment, is usually considered as background data at best only reported in the documentation supporting the database. Such a piece of information could be considered a basis to be further used, and completed, in order to account for resource dissipation in landfills and tailings disposal facilities in future LCA.

Finally, it is noteworthy that in the LCA context, the dissipative flows of resources may need to be allocated between co-products of a multi-functional process, like any other pressures to the environment (e.g. emissions to the environment). The used allocation key may accordingly influence the assessment of resource dissipation along the life cycle of the product or system under study.

### 4.4 Which approaches are used to assess “resource dissipation” in a system in LCA?

#### 4.4.1 Constraints leading to dissipation

The above proposed definition reports “different constraints” as drivers for resource dissipation, with mentioning “technological and economic factors” regarding the “distinction between dissipative and non-dissipative flows of resources”. The chemical elements composing a resource are not “consumed” nor transformed: whatever the process, the mass of any chemical element composing a resource remains constant. On the contrary, mineral resources can be dissipated when they are rendered inaccessible to future users (by definition of “resource dissipation”). The constraints evoked (e.g. low concentrations and large spatial spreading; complex chemical and mineralogical compositions; etc.) imply technological limitations (absence of a suitable technology to process the flows and to make them further usable) and/or economic limitations (technology not economically viable) within the timeframe of the assessment, even when the form and mass of the original resource remains constant in the system under study (e.g. rare earths elements in permanent magnets of waste Hard Disk Drives; Thiébaud et al., 2018). Moreover, technological and/or economic limitations may be dependent on the localization of the potentially dissipative flows (e.g. landfill mining being/becoming technologically feasible and economically viable in some geographical contexts only). Accordingly the geographical representativeness of the model to account for resource dissipation in LCA should be consistent with that generally used to model the LCI system, which depends on the goal of the study and its intended applications (as in the case of the timeframe).

#### 4.4.2 Parameters and thresholds to account for these constraints in LCA

In the reviewed publications, most authors define a set of flows that they consider “dissipative” *per se*, whereas some others have mentioned/applied several parameters to assess dissipative flows (concentrations, mineralogy, particle size, recovery costs and statistical entropy parameters) as more advanced approaches. However, existing LCI datasets are currently not featured to neither of the two accounting approaches. Firstly, exchanges of products within the technosphere include e.g. flows to landfills or to tailings while elementary flows from the technosphere to the environment include e.g. emissions to air, water and soil. Nevertheless, none of these exchanges are currently referred to as “dissipative” in LCI databases. Moreover, the parameters mentioned and even sometimes used in the literature to assess dissipative flows are also absent from LCI databases. The latter could therefore be completed and adapted with such types of information and parameters, yet by means of probably significant efforts. In the meantime further research will be necessary to assess their relevance, including the potential setting of thresholds beyond which one may consider a flow as dissipative.

## 5 Conclusions and potential way forward

The concept of resource dissipation has gained increasing interest in the last two decades in life-cycle based studies. This article shows that while most definitions in published studies intend to capture the difficulty/impossibility to recover a resource, there are different understandings of when a resource is actually difficult or impossible to be recovered and should accordingly be considered “dissipated”. Firstly several authors refer to temporal aspects in their definitions, or as complements to their definitions. However in most cases no temporal aspect is referred to; and when referred to, no given (valued) temporal perspective is explicitly mentioned. Moreover most publications account more or less explicitly for dissipative flows to (or within) at least one of the three following compartments: environment (which relates to what is usually called “emissions to the environment” in MFA and LCA studies), final waste disposal facilities (in technosphere), and products in use (in technosphere). Finally, in order to quantify dissipative flows in the system under study, most authors define a set of flows that they consider “dissipative”, and then calculate the corresponding masses based on different types of data (statistics, process data, assumptions, etc.).

In the absence of a commonly agreed definition of the concept of resource dissipation and of a consistent implementation, its application in LCA is still at a standstill today. In this article, we propose a comprehensive definition for this concept, building from the literature review, and we then discuss this definition with respect to its potential implementation in LCA considering today’s existing datasets and best practices. The LCA framework overall appears well suited to account for resource dissipation. In particular, current LCI datasets cover the flows to the three main compartments of dissipation as distinguished in the literature. However, major challenges are still faced before resource dissipation can actually be routinely, and precisely, assessed. No flows are currently referred to as “dissipative” in LCI datasets, but the latter and their supporting information documents are sources of information that could be further used and completed to potentially account for dissipative resource flows. Moreover the addition of new parameters to evaluate dissipation (e.g. concentrations, mineralogy or entropy, as sometimes mentioned in the literature of life-cycle-based studies) would probably require significant efforts in the context of LCI databases.

These challenges may be overcome, for example, through several steps in a sort of “research agenda” from short-term to long-term. In the short-term, existing LCI databases may be used considering simplified approaches. As a first simplification, one may consider calculating the difference between the amounts of resources extracted and recycled at the level of the whole life cycle, as a proxy for the total amount of resources dissipated. Yet, by definition, this is a simplification that does not allow to assess the contributions of the different life cycle stages or processes to the total dissipation along the whole life cycle. Another simplification could be to flag some flows (to the environment, products in use or waste disposal facilities) as dissipative *per se*, as done so far in some MFA studies (e.g. considering that emissions of metals to the environment or flows of metals to landfills are entirely dissipative and accordingly flagged as such in the LCI). These simplifications could be defined and shared to be systematically applied in LCA. Such an approximation may be particularly consistent for some flows, such as in the case of resources dissipated to the environment, which are dispersed in very low concentrations and represent small, poor-value, deposits. In parallel, new possible approaches to quantify dissipative flows will need to be tested, with application to case studies. This implies, in particular, testing and analyzing several different approaches with respect to the temporal perspective (short, mid or long-term perspectives), the parameters and the thresholds set to quantify resource dissipation.

In the mid-term, these case studies may enable to evaluate the soundness of such approaches, including their robustness and the potentiality for their generalization (for example regarding the feasibility of new data collection to complement existing LCI databases). Additionally, these case studies may foster discussions towards reaching a common understanding on the concept of resource dissipation and its related features, which is essential prior to any large-scale implementation in LCA. Methods to characterize the impact of resource dissipation will need to be developed and tested as well. So far, existing life-cycle-based studies have focused on the assessment of quantities of resources dissipated, primarily in mass units. Mineral resource dissipation reduces “the potential to make use of the value that mineral resources can hold for humans in the technosphere”. This represents the safeguard subject for “mineral resources” within the area of protection “natural resources” as defined by the United Nations Environment Life Cycle Initiative task force (Berger et al., 2020). The quantification of this damage in LCIA would further require either the use of existing characterization models (e.g. the ADP) or the development of new impact characterization models.

Finally, in the long-term, large-scale changes of LCI databases may be required (e.g. updating the existing datasets with new data related to dissipation) considering one or several of the approaches identified as relevant in the “mid-term step”. These changes in LCI datasets will eventually offer the possibility for LCA practitioners to use proper background data in their modelling, and accordingly to systematically account for abiotic resource dissipation in LCA.

## Funding

The authors would like to acknowledge the financial support of the Directorate General for the Environment (DG ENV) in the framework of the Administrative Arrangement on Technical Support for Environmental Footprint and Life Cycle Data Network (EFME3) (07.0201/2015/704456/SER/ENV.AI).

## CRediT authorship contribution statement

**Antoine Beylot:** Conceptualization, Methodology, Formal analysis, Writing - original draft. **Fulvio Ardente:** Conceptualization, Methodology, Writing - review & editing. **Serenella Sala:** Conceptualization, Writing - review & editing, Supervision. **Luca Zampori:** Conceptualization, Methodology, Writing - review & editing.

## Declaration of Competing Interest

The authors declare that they have no known competing financial interests or personal relationships that could have appeared to influence the work reported in this paper.
