# Supplementary material for: Accounting for the dissipation of abiotic resources in LCA: Status, key challenges and potential way forward
Source: Resour Conserv Recycl. 2020 Jun;157:104748. doi: 10.1016/j.resconrec.2020.104748 (PMC7224519; doi:10.1016/j.resconrec.2020.104748)
Supplement: Supplementary file 1 [file mmc1.docx]

**Accounting for the dissipation of resources in LCA: status, key challenges and potential way forward**

**-**

**SI document 1**

Table (A)1 Definitions for ‘natural resources’ (non-exhaustive list from the literature; Ardente et al., 2019)

| *Definition and reference* |
| --- |
| Natural resources can be defined as materials occurring in nature used and transformed by ecosystems and humans, as studied by ecology. (Odum & Odum, 1971) |
| Natural resources are natural assets (raw materials) occurring in nature that can be used for economic production or consumption. (UN, 1997) |
| A resource is an essential input to the economic process. Resources may be material or immaterial (e.g. information) and material resources may be of natural origin or man-made. Services provided by nature (e.g. `assimilative capacity’) are also sometimes called resources. (Ayres, 2000) |
| Natural resources are objects of nature which are extracted by man from nature and taken as useful input to man-controlled processes, mostly economic processes. (Udo de Haes et al., 2002) |
| Natural resources include both the raw materials necessary for most human activities and the different environmental media, such as air, water and soil, which sustain life on our planet. (EC, 2003) |
| Resources are the backbone of every economy and provide two basic functions – raw materials for production of goods and services, and environmental services. (Mensah & Carmago Castro, 2004) |
| Natural resources pertains to materials that are extracted, harvested, or otherwise obtained from the environment for beneficial use by humans. (Bare and Gloria, 2006) |
| Natural resources can be defined as natural assets or endowments from which we derive value (utility). A broad definition would include environmental assets such as wilderness which, while they can be destroyed by human activity, do not have to be consumed in order to have value. (Hatcher, 2008) |
| Natural resources are stocks of materials that exist in the natural environment that are both scarce and economically useful in production or consumption, either in their raw state or after a minimal amount of processing. (WTO, 2010) |
| Natural resources provide essential inputs to production […]. Natural resources are also part of the ecosystems that support the provision of services such as climate regulation, flood control, natural habitats, amenities and cultural services that are necessary to develop man-made, human and social capital. (OECD, 2015) |
| Natural resources are the state’s environmental and ecological assets; the land, water, plants and animals that sustain us and enhance our quality of life. (State of the Rhode Island, 2015) |
| Natural resources are defined broadly as the means for human actions and basis of human livelihoods provided by nature […]. They are extended by all ecosystem functions of earth and solar system usable by humans or funding human well-being […] and the extracted raw materials sub-categorised in biotic and abiotic materials. Their value for humanity as living resource-pools embedded in ecosystems or as single resource units is given by provisioning, supporting, cultural and regulating ecosystem - or resource-services. (Holzgreve, 2015) |
| Natural resources are any raw materials (matter or energy) which are not created by humans, but are available to sustain human activities. (Banai, 2016) |
| Natural resources are material and non-material assets occurring in nature that are at some point in time deemed useful for humans. (Sonderegger et al., 2017) |
| Resources — including land, water, air and materials — are seen as parts of the natural world that  can be used in economic activities to produce goods and services. (IRP, 2019) |

**REFERENCES**

Ardente, F., Beylot, A., Zampori, L., 2019. Towards the accounting of resource dissipation in LCA. XIII Conference of Rete Italiana LCA, Rome, 14-15 June 2019.

Ayres, R.U., 2000. Resources, scarcity, growth and the environment. 2000/31/EPS/CMER.

Banai, A, 2016. Sovereignty over natural resources and its implications for climate justice. WIREs Clim Change.

Bare, J. C., Gloria, T. P., 2006. Critical analysis of the mathematical relationships and comprehensiveness of life cycle impact assessment approaches. Environ. Sci. Technol., 40, 1104−1113.

European Commission, 2003. Towards a Thematic Strategy on the Sustainable Use of Natural Resources. COM(2003) 572 final.

Hatcher, A. 2008. Introduction To Natural Resource Economics - Lecture 1 Natural resource exploitation: basic concepts. University of Portsmouth.

Holzgreve, S., 2015. Defining Natural Resources as Common Goods. Leuphana University of Lüneburg. Dissertation thesis No 3015127.

IRP (2019). Global Resources Outlook 2019: Natural Resources for the Future We Want. Oberle, B., Bringezu, S., Hatfield-Dodds, S., Hellweg, S., Schandl, H., Clement, J., and Cabernard, L., Che, N., Chen, D., Droz-Georget , H., Ekins, P., Fischer-Kowalski, M., Flörke, M., Frank, S., Froemelt , A., Geschke, A., Haupt , M., Havlik, P., Hüfner, R., Lenzen, M., Lieber, M., Liu, B., Lu, Y., Lutter, S., Mehr , J., Miatto, A., Newth, D., Oberschelp , C., Obersteiner, M., Pfister, S., Piccoli, E., Schaldach, R., Schüngel, J., Sonderegger, T., Sudheshwar, A., Tanikawa, H., van der Voet, E., Walker, C., West, J., Wang, Z., Zhu, B. A Report of the International Resource Panel. United Nations Environment Programme. Nairobi, Kenya.

Mensah, A.M., Carmago Castro, L., 2004. Sustainable resource use & sustainable development: a contradiction?! University of Bonn - ZEF. November 2004.

Odum, E. P., Odum, H. T., Andrews, J., 1971. Fundamentals of Ecology; Saunders: Philadelphia, Vol. 3.

OECD. Material Resources, Productivity and the Environment: Key Findings. OECD Green Growth Studies view. February 12, 2015.

Sonderegger, T., Dewulf, J., Fantke, P., Maia de Souza, D., Pfister, S., Stoessel, F., Verones, F., Vieira, M., Weidema, B., Hellweg, S., 2017. Towards harmonizing natural resources as an area of protection in life cycle impact assessment. Int. J LCA 22(12).

State of the Rhode Island, 2015. The Rhode Island comprehensive planning standards guidance handbook series - guidance handbook #2: planning for natural resources. January 14, 2015.

Udo de Haes, H.A., Finnveden, G., Goedkoop, M., Hertwich, E., Hofstetter, P., Klöpffer, W., Krewitt, W., Lindeijer, E., 2002. Life-cycle impact assessment: striving towards best practice SETAC Press, Florida.

United Nation, 1997. Glossary of Environment Statistics, Studies in Methods, Series F, No. 67, United Nations, New York.

World Trade Organisation. World Trade Report 2010 - Trade in natural resources.
